# Improving functional outcome and quality of life for patients with metastatic lesion of acetabulum undergoing cement augmentation

**DOI:** 10.1097/MD.0000000000017029

**Published:** 2019-09-06

**Authors:** Taiqiang Yan, Zhiqing Zhao, Xiaodong Tang, Wei Guo, Rongli Yang, Shun Tang, Sen Dong

**Affiliations:** Musculoskeletal Tumor Center, Peking University People's Hospital, Xicheng District, Beijing, China.

**Keywords:** acetabular metastasis, percutaneous acetabuloplasty, quality of life, radiofrequency ablation

## Abstract

There is an increased enthusiasm in treating osteolytic metastatic acetabulum via injecting polymethyl-methacrylate (PMMA) as a bone filler to provide pain relief and potential structural support. The aim of this respective study is to determine the function and quality of life improvement after cement acetabuloplasty.

Thirty two patients underwent acetabular cement augmentation between May 2014 and March 2018 were respectively reviewed. Isolated percutaneous acetabuloplasty (PA) was performed in 15 patients (group A) while radiofrequency ablation with PA (RFA-PA) in 12 patients (group B). Together with PA, open reconstructive surgery on ipsilateral femur was performed in another 5 cases (group C). Pre- and posttreatment functional evaluation and quality of life (QoL) assessment were carried out.

The average followup duration was 11.5 (range, 3–36) months. None of major complications occurred. 81.2% (26/32) of patients achieved complete pain relief. Reduction of pain intensity and improvement of functional status achieved significantly differences after treatment (*P* = .00). Significant improvement (*P* = .00) was observed in scales of global QoL and pain-related restrictions in daily activities. Both isolated PA and RFA-PA procedures were equally effective towards the improvement of function and quality of life (*P* > .05). Regarding 5 patients in group C, pain intensity decreased when loading the affected limb and they could walk with crutches or cane.

Bone cement acetabuloplasty is an adequate and effective mini-invasive procedure to relieve pain, restore function, and enhance the quality of life of patients for as long as possible in metastatic patients with short life expectancy. Ipsilateral surgery appears to be safe and well tolerated.

## 1 Introduction

The number of patients suffering from metastatic carcinoma to the pelvis has been increasing, and, osteolytic lesion at weight-bearing dome will cause hip pain and decreased mobility, with a significant degradation of quality of life (QoL).^[1–3]^ In the past decades, health-related QoL has been increasingly recognized as an important outcome measure in clinical decision making, particularly in the setting of advanced disease. Therefore, function and QoL are profoundly important considerations when treating metastatic disease of the acetabulum due to the patient's limited life expectancy.

External beam radiation therapy helps reducing hip pain, however, 20% to 30% of patients reported no pain relief, and also radiation does not improve the mechanical properties of the affected acetabular region.^[4,5]^ When nonoperative treatment options unable to provide pain relief or function restoration, operative intervention should be indicated. However, currently, no specific guidelines exist that provide indications for surgical intervention of acetabular metastases. The Harrington periacetabular reconstructive surgery is technically challenging, and carries a substantial risk of complications, the undesirable effects of this large operation may outweigh the clinical benefits for those patients.^[6,7]^

Percutaneous acetabuloplasty (PA), analogous to vertebroplasty, was first adopted by Cotten et al^[8]^ in 1995, and a few reports thereafter have shown this technique is reliable for the management of acetabular osteolysis in patients who cannot be candidates to major surgery and in patients whom radiotherapy was not effective. Recently, radiofrequency ablation (RFA) has been reported to be used for metastatic bone disease^[4]^ or combined with PA (RFA-PA) in the same procedure.^[9–11]^ We conducted this retrospective study and hypothesized that the combination of RFA and PA has additional merits for local tumor control and produces better clinical outcome compared with PA alone. Moreover, few papers addressed the quality of life improvement after the procedure.^[3]^

## 2 Materials and methods

This study was approved by Review Board/Ethics Committee of the Peking University People's Hospital. Informed consents from all subjects were waived due to the retrospective nature of this study. The medical files of 32 patients with acetabular metastases (Harrington III) treated with PA at our institution between May 2014 and April 2018 were retrospectively reviewed (Table 1).

**Table 1:**
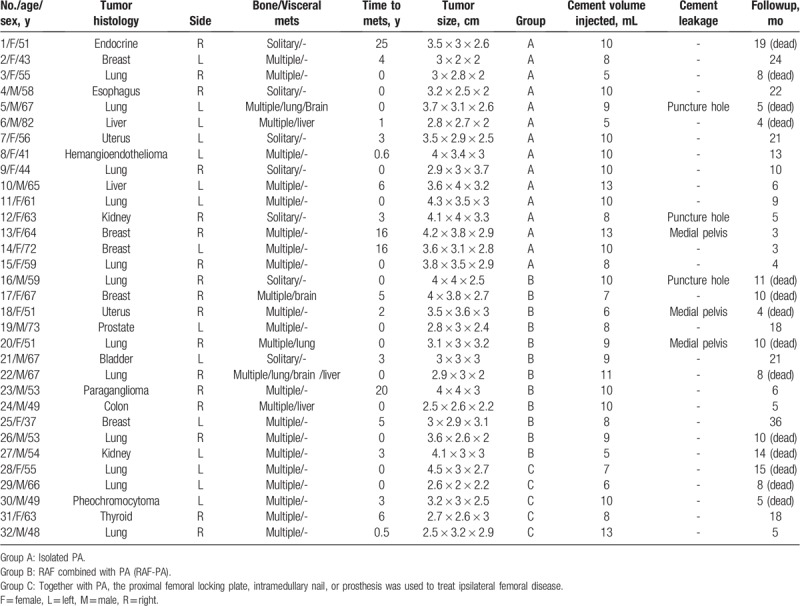
The demographic and clinical data of the study cohort.

The location and extent of the lytic process, the presence of cortical destruction or fracture and the presence of soft-tissue involvement as well as proximity of neurovascular structures were assessed by using computed tomography (CT) and magnetic resonance imaging. Tumor size was calculated on measurement of the largest cross sectional area of a given lesion in coronal, axial, and sagittal planes on 2-mm-slice CT scans. The decision make process was carried out from patient's general condition and life expectancy, starting from a minimally invasive palliative treatment to periacetabular reconstructive surgery. Tables 2 and 3 showed the indications and contraindications of these 2 procedures, respectively.

**Table 2:**
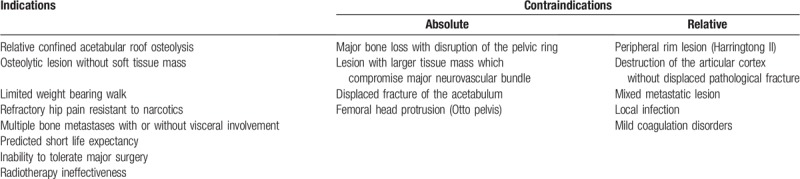
The indications and contraindications to percutaneous acetabuloplasty.

**Table 3:**
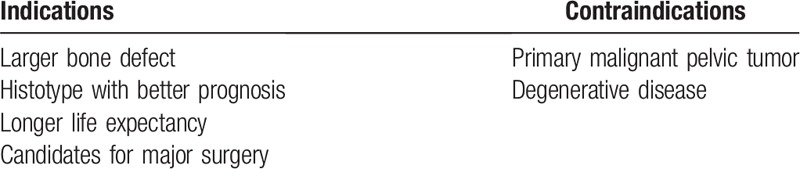
The indications and contraindications to peri-acetabular reconstruction surgery.

All patients were evaluated before and 1, 3, 6, 12 months after treatment regarding pain, general condition, and function related to surgery, by using the Visual Analog Scale (VAS), the Eastern Cooperative Oncology Group performance status (ECOG),^[12]^ and Musculoskeletal Tumor Society score (MSTS),^[13]^ respectively. QoL was assessed using the European Organization for Research and Treatment of Cancer core quality-of-life questionnaire (QLQ-C30),^[14]^ which consists of 5 functional scales (physical, role, emotional, social, and cognitive function), 3 symptom scales (pain, fatigue, and financial difficulties), and a global health and quality-of-life scale. Each health profile is scored on a 100-point scale: a higher score in the functional scales indicates better function while a higher score in the symptom scales reflects a heavier symptomatic burden; a higher score on the global health and quality-of-life scale indicates better health and better QoL.

Fifteen men and 17 women with an average age of 57.6 (range, 37–82) years were included. Eighteen patients had history of cancer and the other 14 patients presented with acetabular metastasis at diagnosis and received biopsy during the same procedure. Twenty-five patients (78.1%) had multiple bone lesions, while 7 with solitary bone lesion. All metastatic acetabular bone lesions with non-small cell lung cancer (12 cases) and breast cancer (5 cases) occurring most frequently. The mean time interval between the diagnosis of lung cancer and the manifestation of the acetabular metastasis was 0.5 (range, 0–6) month, while for breast cancer was 110.4 (range, 48–192) months (*P* = .03). All patients were incapacitated by hip pain. Twenty-three (71.9%) had severe pain requiring continuous use of narcotics, 9 (28.1%) had moderate pain requiring periodic use of narcotics. Ten (31.3%) of them could not walk, 14 (43.7%) needed crutches or a single cane to walk, and the remaining 8 walked without assistive devices.

**Figure 1:**
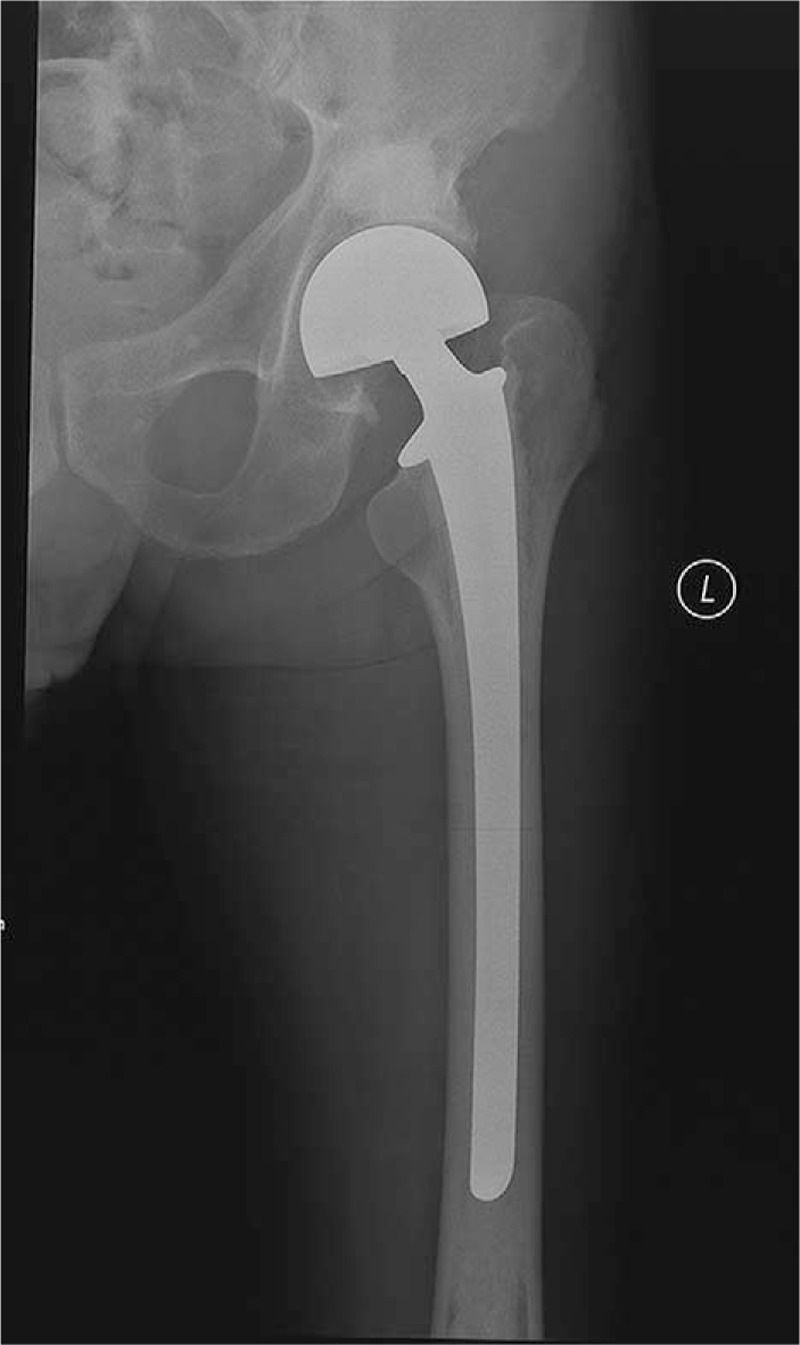
Together with PA, a femoral head prosthesis was used to treat ipsilateral femoral neck metastasis (patient 29). PA = percutaneous acetabuloplasty.

Nonoperative management with protected weight-bearing and analgesics were initially recommended, however, none of patients achieved satisfactory pain relief and function restoration after 1 month or more. Isolated PA was performed in 15 patients (group A), while the RAF-PA in 12 patients (group B). In the other 5 patients (group C), together with PA, the proximal femoral locking plate or intramedullary nail was implanted to prevent impending fracture of ipsilateral femoral diaphysis in 3 patients; a femoral head prosthesis (Fig. 1) or a tumor bipolar endoprosthesis was used to treat ipsilateral femoral neck or intertrochanteric metastasis in 2 patients.

### 2.1 Surgical procedure

In group A, the patients received the procedure under local anesthesia. In group B and group C, general anesthesia with endotracheal intubation was applied.

**Figure 2:**
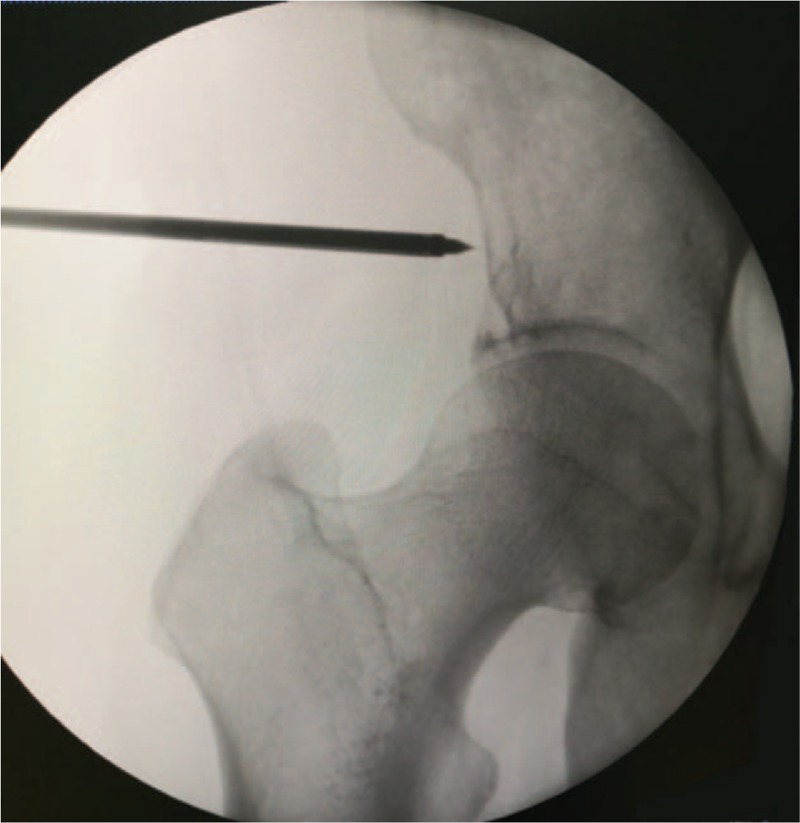
Needle placement into the acetabulum lesion was via lateral approach.

Needle placement into the acetabulum lesion was via lateral approach in order to avoid damage to either the sciatic nerve or femoral artery and vein. We marked the skin entry site at the middle of line between the anterior superior iliac spine and apex of greater trochanter with the patient in supine position, about 2 cm above the acetabular dome for placement of the trocar. A 10-G, 15 cm length needle was advanced towards the lesion (Fig. 2). The correct position of the needle was assessed by anteroposterior (AP) and lateral fluoroscopy views or sometimes, CT guidance.

Ablation was performed with the Tumor Ablation System (ANGIODYNAMICS, RITA Model 1500X; Latham, NY). The procedure was conducted according to the protocol supplied by the equipment manufacturer. Once the target intra-tumoral temperature of 100 °C was obtained, this temperature was maintained 5 to 10 minutes, which was considered as an indicator of adequate thermocoagulation. A single ablation was performed in 5 patients with lesions ≤3 cm in the longest diameter. For large lesions >3 cm in the other 7 patients, the cluster RFA electrode technique (4 needles spaced 5 mm apart) was used.

**Figure 3:**
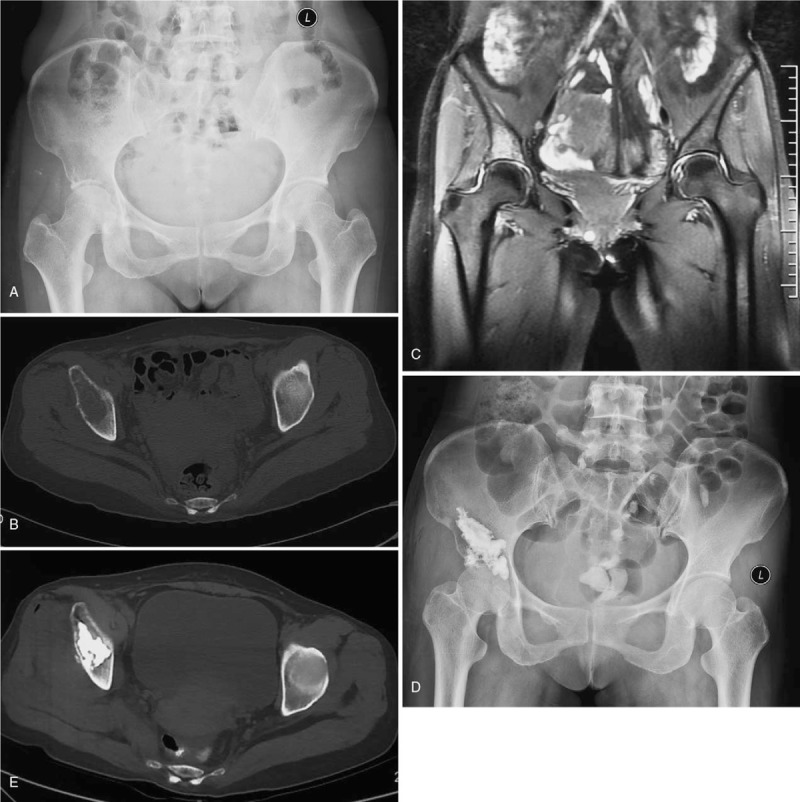
The x-ray film (A), CT scan image (B), and MR image (C) showed the osteolytic lesion at right acetabulum which was histologically confirmed lung cancer (patient 9). The x-ray film (D) and CT scan image (E) indicated that PMMA was injected through the trocar into the lesion, with small cement leak. CT = computed tomography, PMMA = polymethyl methacrylate.

Polymethyl methacrylate (PMMA) was mixed and injected through the trocar into the lesion using a screw injector (Stryker Corp., Kalamazoo, MI) with intermittent fluoroscopy to monitor cement distribution. During the procedure, we mobilized the hip joint just in case of unwanted cement extravasation into the joint space. Although there was no explicit criterion for adequate cement filling within the lesion, in general, we sought to fill the weight bearing surface and at least 50% of the lesion under positive injection pressure. After the procedure, AP and lateral radiographs and CT scans showed a more precise determination of the filling (Fig. 3).

While, in group C, the 5 cases required ipsilateral femur fixation or alloplasty, and the cement was injected using an open surgery method.

### 2.2 Postoperative management and followup

The patients of groups A and B were permitted to walk with partial weight-bearing the next day. Multiplex bisphosphonates were administrated to all patients after the procedure. Adjuvant treatment was administrated in 27 patients (84.3%) according to the primary tumor's histology: systemic chemotherapy or targeted medicine in 5 cases, radiotherapy in 14, and combination therapy in 8 patients. Routine followup evaluation was performed 1, 3, 6, 12 months postoperatively. Each followup evaluation included clinical and imaging studies.

### 2.3 Statistics

Statistical analysis was carried out using SPSS software package version 16.0 (SPSS Inc., Chicago, IL). The comparison of demographic and clinical variables among groups was analyzed by using the independent samples *t* tests and Fisher exact test. Paired-samples *t* tests were applied to compare the pre- and posttreatment scores of VAS, ECOG, MSTS, and each domain of QLQ-C30. Significance was set at *P* < .05.

## 3 Results

Mechanical stabilization of the acetabular lesion was achieved in all patients. Five patients (2 in PA and 3 in RFA-PA) experienced transitory worsening in pain following injection, and resolved spontaneously within 48 hours. The average volume of PMMA injection was 9 (range, 5–13) mL. Small cement leaks into soft tissue due to cortical osteolysis or puncture holes were detected in 6 patients (18.7%) without clinical symptoms. Other potential complications including pulmonary embolism, vascular or nervous injury, hip joint cement leakage, and infection were not observed. Complete pain regression was achieved in 26 patients (81.2%) after treatment, and pain reduction in 6 patients (19%) who required periodic use of narcotics, and the pain did not increase when loading the affected limb.

**Figure 4:**
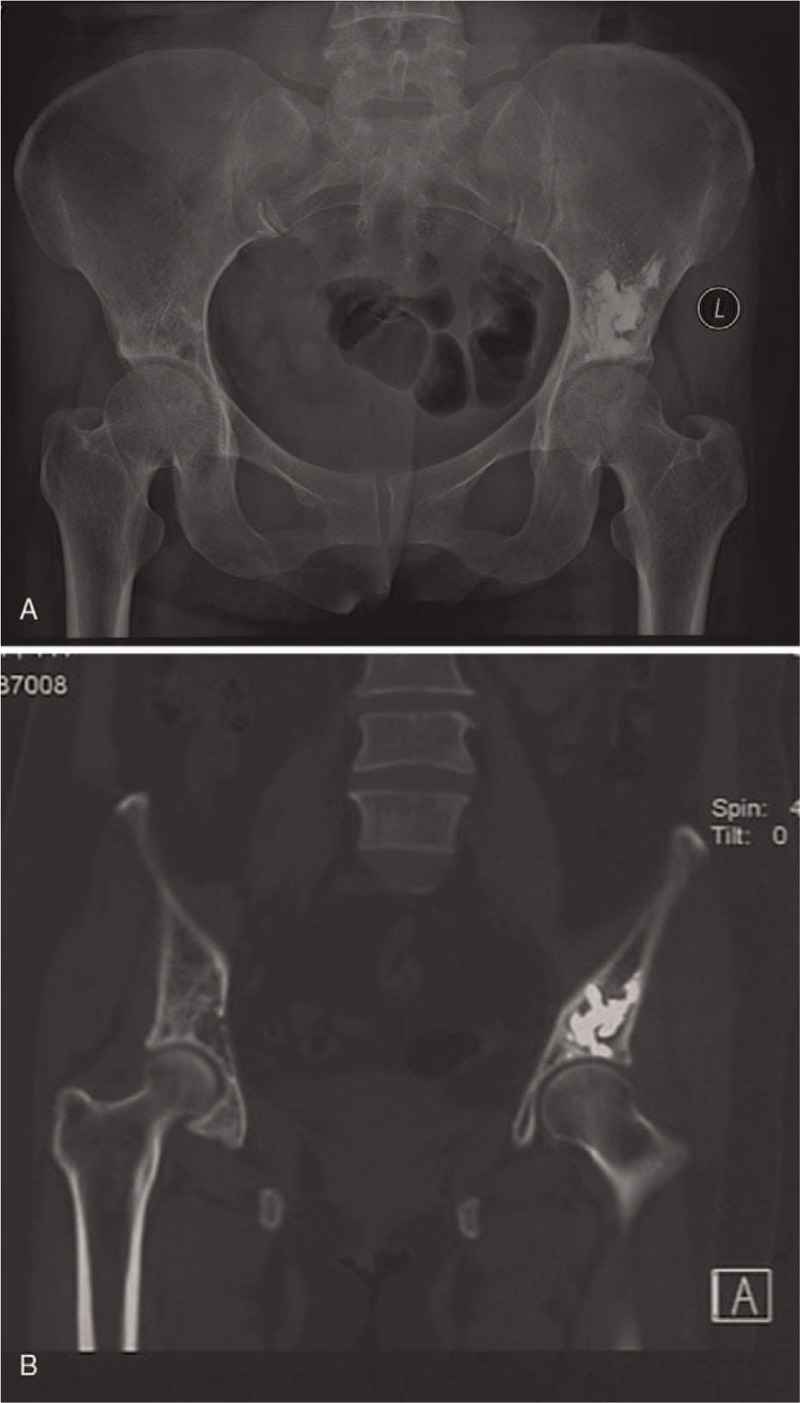
The x-ray film (A) and CT scan image (B) taken 24 months after isolated PA treatment did not reveal osteolysis or bone cement dislocation in the area of cementation (patient 2). PA = percutaneous acetabuloplasty.

**Figure 5:**
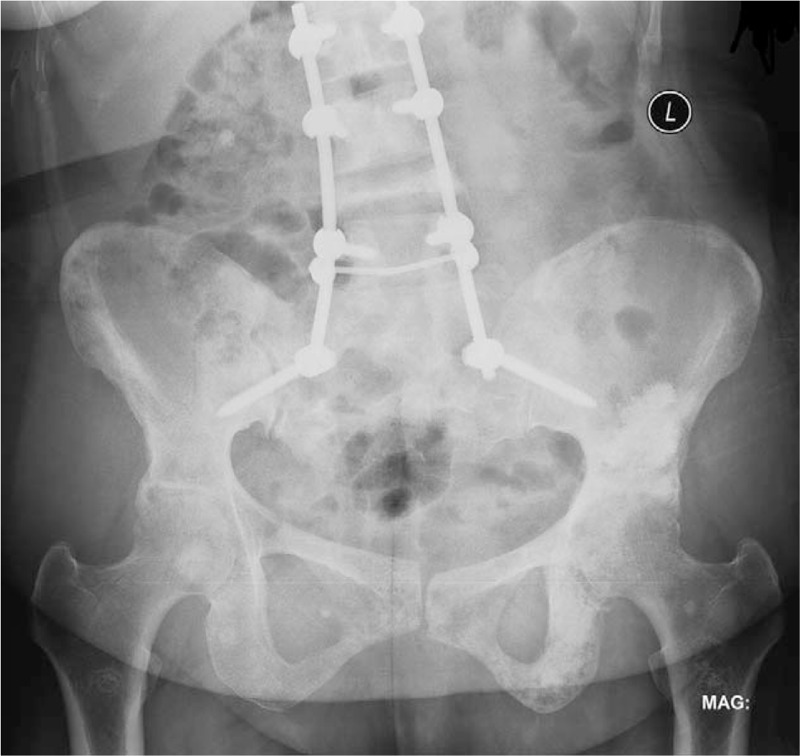
The patient underwent further reconstructive operation on lumbar metastatic lesion 12 months after RFA-PA procedure in her left acetabulum, however, antero-posterior pelvic radiographs taken at 36 months followup did not show any change at the area of cement augmentation (patient 25). PA = percutaneous acetabuloplasty, RFA = radiofrequency ablation.

Mean followup duration was 11.5 (range, 3–36) months. During the followup, 9 patients (patients 8, 10, 14, 20, 22, 25, 26, 30, and 31) received further operation at tibia, humerus, clavicle, thoracic, or lumber spine, respectively. All patients were alive at 3 months; at 6 months 4 patients died of disease; at 12 months, another 7 patients died of disease; after 12 months, another 3 patients were dead. Eight of 12 patients with lung cancer metastases died at the mean of 9.5 (range, 5–15) months after treatment despite of systematic chemotherapy or targeted medicine, while only 1 of 5 patients with breast cancer died of brain metastasis at 11 months after PA (*P* = .13). Posttreatment radiographs did not reveal osteolysis in the area of cementation, bone cement dislocation, or loosening within the acetabular bone (Figs. 4 and 5). Pathological fracture within the strengthened acetabulum was not found. No one was reverted to secondary open reconstruction surgery.

### 3.1 Pain, function, and QoL evaluation in groups A and B

There was a statistically decrease in VAS score (as shown in Table 4) after treatment. The pain intensity measured by VAS decreased from 7.44 preoperatively to 3.85 at 1 month (27 cases), 3.70 at 3 months (27 cases), 4.00 at 6 months (19 cases), and 3.67 at 12 months (9 cases). The ECOG scores showed slightly improvement after treatment, improving from 2.74 prior to surgery to 2.63 (1 month), 2.62 (3 month), 2.57 (6 month), and 2.22 (12 month). One patient could not walk, 7 walked with 2 crutches, 9 with a single cane, and others walked unaided. A similar trend regarding MSTS score was observed, also showed a marked improvement after treatment, improving from 37.8% preoperatively to 47%, 50%, 51.2%, and 55.6% at 1 to 12 months, respectively.

**Table 4:**
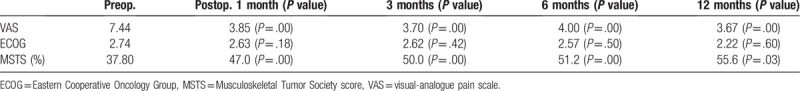
Pre- and postoperative scores of VAS, ECOG, and MSTS.

The QoL improvement of the patients after treatment was also evident. Most patients restored the ability of carrying out standard daily activities. The mean functional and symptom scores of QLQ-C30 before and after treatment were shown in Table 5. Paired-samples *t* test examination of the posttreatment score of 46.96 showed a significant improvement in QoL from the preoperative score of 36.36 (*P* = .00). With regard to physical function, although more restrictions were complained in scales of long distance walk and heavy luggage carry, there was slightly improvement after treatment compared with pretreatment score (44.54 vs 40.90, *P* = .05). Restrictions in the ability to perform hobbies or other daily activities (role function) were reported to be a mean score of 31.05, a slightly difference to 27.27 pretreatment (*P* = .06). With respect to other functional scales (emotional, cognitive, and social functioning), no significant difference was found after treatment. A minor improvement (*P* = .08) was observed in the scale of sleeping component due to pain relief after treatment. Significant improvement (49.23 vs 78.03, *P* = .00) was seen in the symptom domains of pain and pain-related restrictions in daily activities. Also, the scores of financial difficulties were 62.12 pretreatment and 68.18 posttreatment, and the difference was more pronounced (*P* = .04).

**Table 5:**
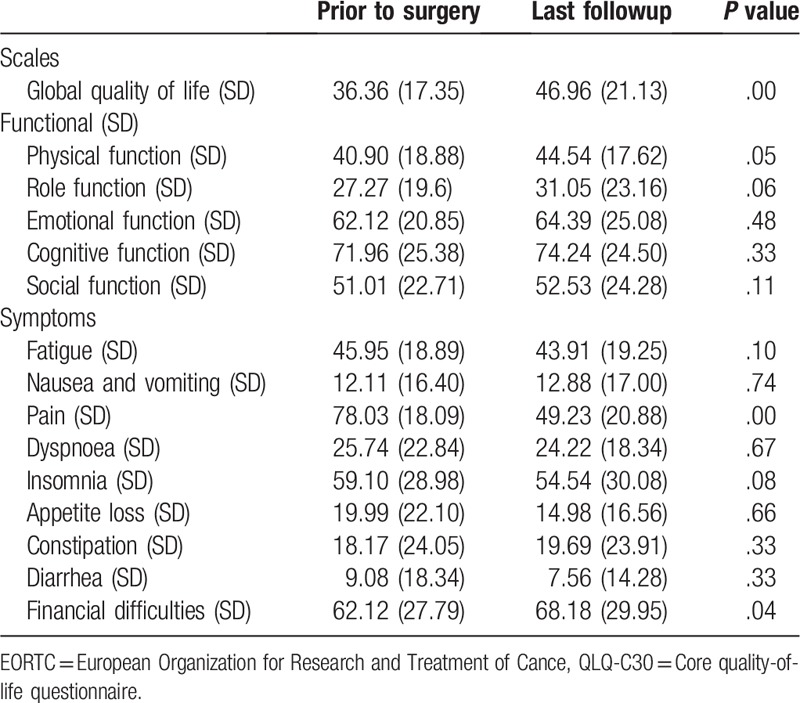
Descriptive analysis of HRQOL scores (means, standard deviation) of EORTC QLQ-C30 before treatment and the last followup for all patients.

### 3.2 Comparison between isolated PA and RFA-PA

To detect whether the application of RFA has added benefits, we compared VAS, ECOG, MSTS, and global QoL between groups A and B. Groups A and B were similar regarding the age, the median size of bone lesion, the cement injection volume, and the average followup period (Table 5). Also, 11 of 15 (73.3%) in group A, 9 of 12 (75%) in group B received postoperative radiation (*P* = 1.000, Fisher exact test). At baseline, the median scores of VAS, ECOG, MSTS, and QoL were 7.40, 2.66, 38.15%, and 33.98 in group A, while in group B they were 7.50, 2.83, 36.00%, and 39.80, respectively. In terms of pain, limb function, and QoL improvement, the superiority of RFA-PA procedure did not reach significant differences over PA alone, as shown in Table 6.

**Table 6:**
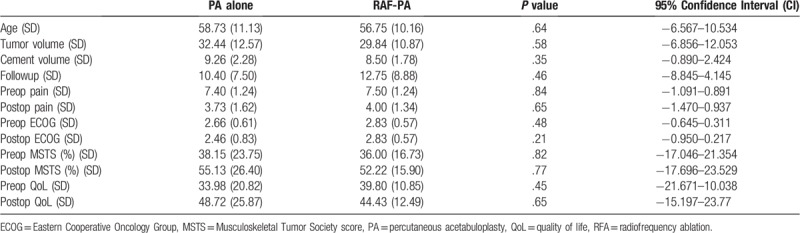
Comparison data between PA alone and RAF-PA at last followup after operation.

### 3.3 The evaluation of patients in group C

For 5 patients in group C, the mean VAS, ECOG, and MSTS score were 8.0, 3.2, and 30% respectively before the surgery, and 4.2, 3.0, and 34% at 1 month, 3.8, 2.8, and 38% at 3 months, 4.3, 3.0, and 41% at 6 months, and 5.5, 3.0, and 30% at 12 months after treatment, respectively. Three patients could walk with 1 crutch or cane and 2 patients with 2 crutches. Regarding the improvement of health-related QoL, the following mean scores were recorded at last followup was 35.01 compared with 29.98 preoperatively.

## 4 Discussion

Our center previously presented the data of 46 patients who underwent Harrington reconstructive surgery for peri-acetabular metastases in 2011.^[2]^ There was a statistically significant improvement of QoL after surgery (*P* = .00), and pain reduction was also significantly changed (*P* = .00). In addition, 14 of 46 patients underwent curative en-bloc resection did not have survival benefit compared with curettage (*P* = .61). Another study conducted by Ruggieri et al^[15]^ also did not demonstrate any statistically significant difference in oncological outcome between the patients after wide resection and those after intralesional resection. In line with this, we have explored alternative methods of providing structural reinforcement which are less invasive and less prone to the major morbidities of open reconstruction in recent years.

Some limitations in our study included its retrospective nature, small population size, the heterogeneity of the histotype, multiple bone lesions, and lack of adjustments for other potential confounders such as pre-procedure analgesic use and concurrent radiotherapy/chemotherapy. However, we included only lesions located at acetabular dome (Harrington III) without soft tissue mass/neurovascular bundle compression in this study to make the evaluation parameters comparable. Lesions in the ilium, sacrum, medial wall (Harrington II), or involving the entire hemipelvis were excluded. The mean duration of followup was only 11.5 months, which is within the range reported by other series, possibly it is due to 37.5% (12/32) of the patients were metastatic lung carcinoma.

As we expected, cement injection succeeded in achieving satisfactory pain relief in this cohort of patients (81%), which was similar to previous reports.^[16–19]^ Maccauro et al^[17]^ have performed PA in 25 patients, complete pain regression was recorded in the majority of patients. In Gupta's study,^[16]^ 11 patients with lytic metastases to acetabulum underwent percutaneous acetabuloplasty, 81.8% of them achieved complete pain regression. Moser et al^[19]^ observed a therapeutic response in 78.1% of 44 pelvic metastatic lesions (30 located at acetabulum). Two main factors, we believe, may account for pain relief. One is the exothermic reaction developed during the polymerization of the cement mediates tumor eradication or neurolysis of pain fibers. The another is that PMMA provides structural buttressing of the weight-bearing portion of the joint even with subtotal lesional filling, resulting in improved biomechanics.

We did not notice that patients’ general condition significantly improved after the treatment (Table 2), not the same as Maccauro and Colman reported.^[17,20]^ It is understandable, because 25 patients (78.1%) in this study had multiple bone lesions (6 of 25 with concomitant visceral involvement), obviously, the general condition of our patients was poorer and 9 patients received further operations for other bone metastases. Therefore, the general condition improvement evaluated by ECOG index was not significant. However, the functional performance status assessed by MSTS score was significantly improved after the treatment (as shown in Table 4). We would like to emphasize that this was in agreement with the literature justify its treatment.^[21]^

In terms of improvement of QoL, few studies have addressed this issue. Scaramuzzo et al^[3]^ had analyzed ECOG, QLQ-C30, and The MOS 36-Item Short From Health Survey in 20 patients after percutaneous injection of PMMA. A marked improvement in physical condition and global QoL was found in the first 6 months, but a worsening of the QoL was observed in the next 6 months due to deterioration of general condition and progression of the primary pathology. Guzik^[22]^ performed bone cement augmentation in 21 patients, of whom 9 had cement injection percutaneously, while 12 patients required proximal femur resection alloplasty. The mean Karnofsky functional status scores were 52.5 before the surgery and 71.8 after the procedure, respectively. The mean postoperative Harris hip score was 94. In our study, the patients underwent cement augmentation achieved a significant improvement in QoL. Paired-samples *t* test examination of QoL scores of QLQ-C30 showed significant improvement in QoL at last followup for all patients. Physical function, role function, and sleeping were found to be slightly improved due to pain relief after treatment. Significant improvement (*P* = .00) was seen in the scales of symptom domains of pain and pain-related restrictions in daily activities. Interestingly, we observed that the change of financial difficulties was more pronounced (*P* = .04) in this series, we think it was related to the increasing economic burden of chemotherapy, radiotherapy, or targeted medicine or more operations needed to solve metastasis at other sites.

While RFA and cement acetabuloplasty are independently effective in pain palliation,^[4,16–19,21]^ some studies suggest that the combination of RFA and cement injection may have a synergistic effect on pain management.^[9–11]^ The majority of these studies are single-arm observational studies and there is lack of level 1 evidence supporting increased efficacy in pain management following combined RFA and cement injection. Toyota et al^[9]^ described RFA combined with cementoplasty in 17 patients. Pain relief was achieved in 100% of patients, the mean duration of pain relief was 7.3 months. Wallace et al^[10]^ reported the outcomes of 12 patients who underwent RFA combined with cementoplasty. The median posttreatment pain score was 3, a significant difference compared with pretreatment of 8 (*P* = .00). 73% of patients experienced partial pain relief and no immediate symptomatic complications occurred after treatment. In a retrospective single-center observational study, 55 spinal metastases received combined RFA and vertebroplasty over a median follow-up period of 34 weeks, Wallace et al^[23]^ reported 89% (41/46) and 70% (21/30) radiographic local control rate at 3 months and 1 year post procedure, respectively, despite systemic metastatic disease progression. These preliminary results from combined RFA and cement augmentation were promising. In contrast, Orgera et al^[24]^ performed a randomized controlled trial of 36 consecutive patients with spinal osteolytic lesions secondary to multiple myeloma, reported similar pain scores post procedure in both vertebroplasty alone and combined RFA and vertebroplasty groups (mean VAS scores at baseline 9.3 vs 9.1, at 24 hours 3.0 vs 3.4 [*P* = .33], at 6 weeks post procedure 2.3 vs 2.0 [*P* = .29]). Both groups also had similar analgesic use and functional levels at all time points without any major complications following procedure. They concluded that additional RFA may not provide added benefit in pain management of patients with multiple myeloma and vertebral deposits in the medium term. However, to date, there has been no published trial on the topic of acetabuloplasty in a population of patients with metastatic acetabular osteolytic lesions.

In this study, the information of the 2 procedures was documented, with regard to the sex, the average age, the median volume of bone lesion, the cement injection volume, and the average followup period. There were no significant differences between these 2 groups (Table 6), which may increase its validity of this study. Regarding the reduction of pain intensity, improvements of function and QoL, the safety and efficacy of both procedures in the palliative management of acetabular metastasis were justified. Meanwhile, cement strengthened acetabulum failed was not found in groups A and B. Although RAF-PA procedure did not reach significant differences than sole PA when we compared the scores of VAS, ECOG, MSTS, and QoL (Table 6), the added benefits of RFA in palliation could not be negligible, because of its retrospective design, opportunistic case series of patients managed in a single center. Several reasons have been postulated for the equal effectiveness of 2 procedures. Firstly, we tended to use more cement during positive injection under carefully monitoring with continuous adjustment of the needle direction making the filling of bone cement more efficient. Secondly, previous reports^[4,16–18,21,25]^ also indicated that the exothermic reaction arising from the cement's polymerization is basically the same as that obtained by radiofrequency, therefore, no increase of cytotoxic effect on tumor should be obtained. Finally, the majority of patients had postoperative radiation in this study, radiation after cement injection which may act synergistically to achieve better pain palliation and local tumor control.

It is difficult to decide which parameters must be considered to perform isolated PA or RFA-PA. The combination selection could be the inclination of the surgeon and the blood supply of the tumor, particularly in patients who do not respond to medical therapy for the systemic disease. Therefore, we have to state explicitly here that this study does not currently constitute sufficient evidence to recommend a change in practice, for example, the cessation of radiofrequency ablation treatment. Hence, a randomized prospective comparative assessment of the 2 techniques is urgently requiring.

The contemporary presence of an impending proximal femoral fracture was an indication for acetabular cementoplasty during the same anesthesia, which was also reported in previous reports.^[17,22]^ The clinical and functional results of resection alloplasty on the proximal femur combined with periacetabular cement injection were good. This enables the patients to avoid the risk of hemorrhage, and the problems linked to open acetabular reconstruction (patient 29 and 30). The same considerations were made for combined lesions of the femoral shaft and the acetabulum in another 2 patients (patient 28 and 32) and 1 patient with bilateral femur disease (patient 31).

## 5 Conclusion

The growing number of cases in literature together with our consecutive series support that PA is an effective therapeutic mini-invasive procedure to improve patient's functional performance and health-related QoL in a specific group of patients with minimal complications. PA can also be used for combined lesions of the acetabulum and the ipsilateral femur.

## Acknowledgments

The authors thank Ms Yanchun for assistance with data collection.

## Author contributions

**Conceptualization:** Taiqiang Yan.

**Data curation:** Taiqiang Yan, Zhiqing Zhao, Xiaodong Tang, Rongli Yang, Shun Tang.

**Formal analysis:** Zhiqing Zhao, Sen Dong.

**Supervision:** Wei Guo.

**Writing – original draft:** Taiqiang Yan, Zhiqing Zhao, Xiaodong Tang, Wei Guo, Rongli Yang, Shun Tang, Sen Dong.

**Writing – review & editing:** Taiqiang Yan.
